# Transgenic *Drosophila* to Functionally Validate Fall Armyworm ABCC2 Mutations Conferring Bt Resistance

**DOI:** 10.3390/toxins15060386

**Published:** 2023-06-07

**Authors:** Rafaela Panteleri, Amalia Anthousi, Shane Denecke, Debora Boaventura, Ralf Nauen, John Vontas

**Affiliations:** 1 Department of Biology, University of Crete, Vassilika Vouton, 71409 Heraklion, Greece; rpantele@gmail.com (R.P.); amalia_anthousi@imbb.forth.gr (A.A.); 2 Institute of Molecular Biology and Biotechnology, Foundation for Research and Technology-Hellas, 71409 Heraklion, Greece; shane.denecke@protonmail.com; 3 Department of Pathobiology, University of Pennsylvania, Philadelphia, PA 19104, USA; 4 Bayer AG, Crop Science Division, R&D, Pest Control, 40789 Monheim, Germany; debora.boaventuraehlert@bayer.com; 5 Pesticide Science Laboratory, Department of Crop Science, Agricultural University of Athens, Iera Odos 75, 11855 Athens, Greece

**Keywords:** fall armyworm, Cry toxins, resistance, diagnostics, ABC transporter

## Abstract

The fall armyworm (FAW), *Spodoptera frugiperda* (J.E. Smith; Lepidoptera: Noctuidae) is an invasive agricultural pest with a global distribution, causing major crop losses annually. Its control strategies largely rely on chemical insecticides and transgenic crops expressing *Bacillus thuringiensis* insecticidal proteins (Cry and Vip toxins); however, the development of high resistance poses a significant issue. The ATP-binding cassette transporter C2 (ABCC2) has been linked to Cry toxin pore formation, acting as a receptor of some Cry toxins. Recently detected mutations in the SfABCC2 gene in extracellular loop 4 (ECL4) have been associated with Bt toxin resistance in FAW. In the present study, we expressed the SfABCC2 gene in *Drosophila melanogaster*, a species normally unaffected by the Bt toxins. We demonstrate that susceptibility can be introduced by the ectopic and tissue-specific expression of wildtype SfABCC2. Next, we introduced mutations into ECL4—both individually and in combination—that have been recently described in Brazilian FAW and functionally validated by toxicity bioassays against the foliar Bt product Xentari. Our results provide an efficient demonstration of the suitability of transgenic *Drosophila* for validating FAW ABCC2 resistance mutations in ECL4 against Bt toxins, and potential cross-resistance issues between closely related proteins that use ABCC2.

## 1. Introduction

The fall armyworm (FAW) *Spodoptera frugiperda* is one of the most destructive agricultural pest species, causing enormous annual production losses in major crops such as corn and soybean [1,2,3]. The distribution of FAW has been expanded since 2016 through its rapid global invasion from the tropical and sub-tropical Americas to Africa and the Asia–Pacific area [4,5,6,7]. As a result of the extensive use of chemical insecticides, the FAW has been ranked among the top 15 most insecticide-resistant insect species and has evolved resistance to many different chemical classes [8]. Other control strategies rely on the expression of insecticidal toxins derived from *Bacillus thuringiensis* (Bt)—hereafter referred to as Bt toxins—in transgenic crops including corn, cotton and soybean, or—alternatively—their foliar application as Bt toxin mixtures [4]. *B. thuringiensis* naturally produces crystalline (Cry) proteins during sporulation and the crystals are released at the end of the autolysis process. Bt toxicity is expressed after the crystal structures are solubilized in the alkaline environment of the caterpillar midgut. Here, the protoxin is activated through proteolytic cleavage, and finally triggers pore formation in midgut cells upon oligomerization through interactions with specific midgut receptors [9,10,11,12]. Several proteins have been proposed as Cry toxin receptors, including aminopeptidase N (APN), cadherin (CAD), alkaline phosphatases (ALP) and ATP-binding cassette (ABC) transporters. Bt resistance is typically conferred by the reduced binding of Cry toxins to their specific midgut receptor targets, because of altered expression levels and/or the presence of mutations [13]. Thus, a deeper understanding of these receptors is necessary to properly manage resistance to Bt toxins.

Many studies have pinpointed a major role of ABC transporter subfamily C2 (ABCC2) in mediating toxicity (pore-formation) [14,15,16], including functional proof in *Chloridea virescens* (Lepidoptera: Noctuidae), where a knockout of ABCC2 leads to high levels of Bt (Cry1Ac) resistance [17]. The role of ABCC2 in the FAW in Cry1F toxicity has been confirmed in vivo as well, based on a CRISPR/Cas9-mediated ABCC2 knockout [18]. ABCC2 mutations have also been recently associated with Bt (Cry1F) resistance in *S. frugiperda*—more specifically, a 2 bp insertion (GC) in the ABCC2 gene leading to a premature stop codon and a truncated protein associated with Cry1F resistance in FAW populations from Puerto Rico [19,20]. A more recent study identified alternative mutations in ABCC2 that are functionally linked to Cry1F resistance in Brazilian FAW populations, located in extracellular loop 4 (ECL4) [21]. Boaventura et al. (2020) described three mutated sites existing either in isolation or in combination: a GY deletion (positions 788–789) and a P799K/R amino acid substitution—identified in ECL4—and a G1088D amino acid substitution in the intracellular nucleotide binding domain (NBD). The importance of ECL4 ABCC2 mutations was functionally validated in vitro with recombinantly expressed ABCC2 variants in insect-cell lines, followed by the evaluation of their responsiveness against Cry1 toxins [21,22]. 

In the present, brief study, we aimed to design transgenic *Drosophila* lines expressing FAW ABCC2 for employment as a model for investigating Bt toxin efficacy and resistance. We ectopically expressed several alleles of the *S. frugiperda* ABCC2 gene, wild type susceptible variants and variants carrying previously described resistance mutations in isolation (GY deletion or P799K) or in combination (GY deletion and P799K), and assessed the susceptibility of the transgenic *Drosophila* lines against a commercial sprayable Bt formulation as a first step in toxicity bioassays.

## 2. Results

### 2.1. Expression of SfABCC2 in the Midgut of D. melanogaster Leads to Susceptibility against Bt Toxins

**Figure 1:**
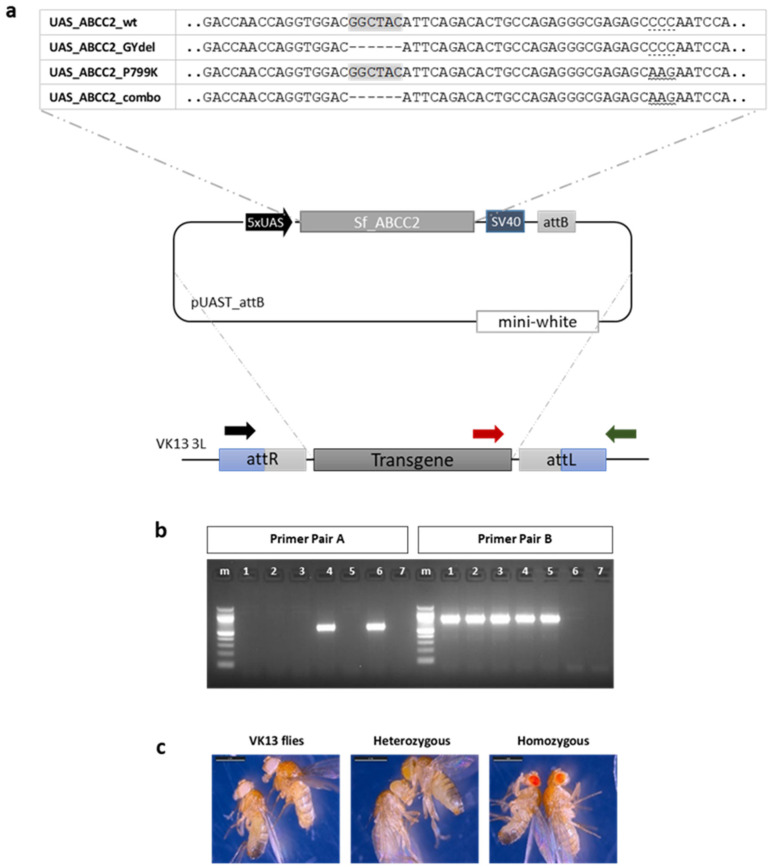
Experimental flow of the current study. (**a**) The wild type allele of SfABCC2 and the mutant alleles (bearing the GY deletion and P799K substitution, alone and in combination) were cloned into pUAST-attB vectors. The four recombinant plasmids were injected into VK13 Drosophila embryos, which carry an attP landing site on the 3rd chromosome. Injected G0 flies were outcrossed with VK13 adult flies and G1 progeny was screened for w+ phenotypes, indicating the integration of the plasmid. The homozygous fly lines were established after a series of crosses. (**b**) PCR screening followed to test integration into the transgenic fly lines. Primer pair A (black and green arrows—3xP3_RFP_F and VK13_R) gives product when integration has not occurred, whereas Primer pair B (red and green arrows—SV40_F and VK13_R) gives product when integration has occurred. m: molecular weight marker, 1: ABCC2_GYdel, 2: ABCC2_P799K, 3: ABCC2_combo, 4: ABCC2_GYdel/+ (heterozygous), 5: ABCC2, 6: VK13, 7: no template control (NTC). (**c**) Eye phenotype of the VK13 line, the heterozygous state after transgene integration and the homozygous state.

**Figure 2:**
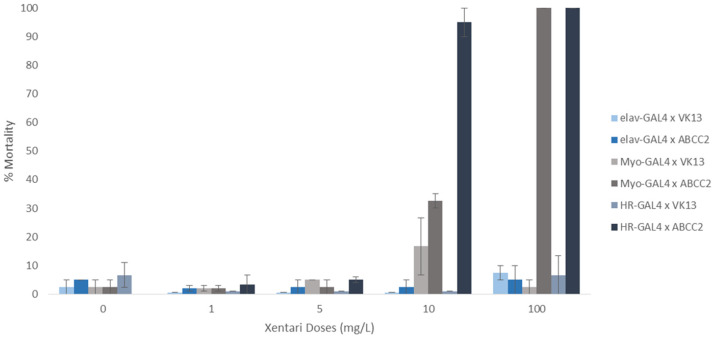
Discriminating dose larval bioassay. Comparison of the mortality (±SEM) at different Xentari doses of flies expressing the SfABCC2 transporter in different tissues (x ABCC2) and others not bearing the transporter (x VK13). ELAV-GAL4 is a neuronal cell specific driver, Myo-Gal4 drives expression in the midgut cells, while HR-GAL4 drives expression in the midgut, Malpighian tubules and fat body. Flies that do not carry the transporter (progeny of elav-GAL4 × VK13, Myo-GAL4 × VK13 and HR-GAL4 × VK13) are not affected by the Cry toxin mixture. The expression of he SfABCC2 transporter in the midgut renders the fly susceptible to the Cry toxin mixture, whereas the flies expressing it in their neuronal cells are not affected by the toxins—even in concentrations as high as 100 mg/L.

The ABCC2 gene of *S. frugiperda* was successfully introduced into Drosophila’s genome using the phiC31 integrase system. The integration was verified by PCR with two different primer pairs (Figure 1). Larval feeding bioassays were then conducted using the commercially available Bt toxin mixture Xentari, to examine the phenotype driven by the expression of SfABCC2 in *Drosophila* in either neurons (ELAV-GAL4), the midgut (MYO-GAL4) or a combination of the midgut, Malpighian tubules and fat body (HR-GAL4). As expected, flies that did not express any SfABCC2 were unaffected by any dosage of Xentari, regardless of which driver line was tested (Figure 2). Similarly, expression of SfABCC2 in the central nervous system (ELAV-GAL4) did not render flies susceptible to Xentari (Figure 2). However, expression of SfABCC2 either specifically in the midgut or in the midgut in combination with other tissues (MYO-GAL4 or HR-GAL4) caused significant mortality in *Drosophila* larvae at 10 and 100 ppm. The more discriminatory dose of 10 ppm showed a substantially larger effect from SfABCC2 expression using the HR-GAL4 driver. 

### 2.2. Mutations in the ABCC2 Transporter Gene Confer Resistance to Xentari in Transgenic Flies

Several fly lines bearing mutations that are associated with resistance to Cry toxins in the SfABCC2 gene were generated using the phiC31 integrase system. Specifically, a line bearing a GY deletion in the SfABCC2, a line bearing the P799K substitution in SfABCC2 and a line which combines the two mutations were generated. Crossing these lines with the driver HR-GAL4 led to a substantial and synergistic contribution of each mutation to resistance (Table 1). Flies expressing SfABCC2 with the GY deletion and the P799K substitution show rather low, but significant resistance to Xentari in comparison to flies expressing wildtype SfABCC2 (RR 2.88-fold for the GY deletion and 4.69-fold for the P799K substitution). The combination of these two alterations in the gene of SfABCC2 led to higher Xentari resistance levels (RR: 15.2-fold).

## 3. Discussion

In this study, we generated transgenic fly lines expressing FAW ABCC2 variants, allowing an investigation of the effects of otherwise inactive Bt toxins against the model insect *D. melanogaster*. We quantified the contribution of two distinct mutations in the ABCC2 gene of *S. frugiperda* using the transgenic *D. melanogaster* against the commercial Bt spray product Xentari, as it includes Cry1A proteins that have been shown to be affected by ABCC2 mutations [13]. Xentari is a mixture of four Bt insecticidal proteins, which is advantageous for pest control, but has limitations over individual Bt toxins when testing the specific impact of mutations. Prior studies have also made use of *D. melanogaster* to investigate the role of ABCC2 in Bt toxicology, e.g., by ectopically expressing ABCC2 from the diamondback moth *Plutella xylostella* and demonstrating fly sensitivity against Cry1Ac [23]. Likewise, the expression of ABCC2 from the silkworm *Bombyx mori* conferred sensitivity to Cry1Ab in transgenic flies [24]. Here, we attempted to extend this model by firstly testing another ABCC2 orthologue (SfABCC2), but also by exploring the tissue-specific expression of ABCC2-mediated sensitivity. While—as expected—brain-specific ABCC2 expression (ELAV-GAL4) had no effect on *Drosophila* towards the Bt product Xentari, both MYO-GAL4 and HR-GAL4-driven ABCC2 expression conferred sensitivity, with HR-GAL4 having a larger effect. While it is possible that the addition of tubule and fat body expression of ABCC2 in the HR-GAL4 line is responsible, we find this unlikely, given the difficulty of large proteins crossing the midgut epithelial barrier [25]. A more likely explanation would be a difference in the expression strength of these two driver lines in the midgut. Future studies are warranted to systematically test how the location and strength of FAW ABCC2 expression influences the toxicity of individual Cry toxins in the Drosophila system. 

Apart from model exploration, this study much more directly quantified the impact of two SfABCC2 mutations on a mixture of Cry toxins in Xentari—a commercial Bt formulation for foliar application often less affected by cross-resistance issues in Bt resistant strains, as shown for the FAW [26]. Previous work identified two mutations in ECL4 of FAW ABCC2, and both mutations were functionally validated in vitro and shown to confer Cry1F resistance to Sf9 cells expressing mutated ABCC2 when compared to wildtype ABCC2 [21,22]. The data presented here extends the validation to an in vivo system, transgenic *Drosophila* ectopically expressing pest genes, which has been recently reviewed [27]. Indeed, we successfully modified *Drosophila* with wildtype and mutated SfABCC2 variants and confirmed—for the first time in vivo and in a defined genetic background—that both mutations can confer low, but significant levels of resistance to Xentari, but that their combination triggers a significantly higher level of resistance. Previous work [21] analyzed 30 Brazilian FAW field samples by pooled population sequencing, and the predominant ABCC2 mutation was a GY deletion (position 788–789) in ECL4; however, the authors also detected a number of rare alleles which disrupted residues in ECL4, between sites 783–799. Some of these alleles were recently tested and validated in vitro by expressing mutant ABCC2 in Sf9 cells [22]. The in vivo system introduced here displays an excellent starting point to further explore the importance and involvement of ECL4 as well as other ECLs in mediating toxicity (pore formation) by testing individual Bt toxins rather than a mixture, as carried out here for proof of concept.

The fact that the combined mutations of SfABCC2 confer higher levels of Bt resistance than the individual mutation in transgenic flies further highlights a phenomenon often described in small molecule pesticide resistance, whereby distinct resistance alleles combine to generate resistance levels greater than the sum of their individual parts (synergism). This has been recently demonstrated functionally [28], and explains part of the gap that exists between the relatively small levels of resistance observed in laboratory settings compared to the field. 

The ability to rapidly test such mutations in an in vivo system makes *Drosophila* a powerful model, sitting at the interface between cell-based in vitro models and the gold-standard CRISPR/Cas9-based modifications in the pests themselves. Although cell-based in vitro studies have demonstrated the predictive value of resistance levels [22], it is difficult to precisely relate these metrics to in vivo resistance ratios that would be the most relevant from an applied perspective. On the other hand, making CRISPR mutations directly in lepidopteran pests such as *Helicoverpa armigera* (Lepidoptera: Noctuidae) has also been used to measure the impact of Bt resistance alleles [29], but these studies are very time-consuming and of low throughput. Here, *Drosophila* might serve as an alternative model. However, it is important to note that the results may not fully represent the natural response of the FAW to Bt toxins. Inherent physiological or genetic differences between Drosophila and the FAW might affect the interpretation and extrapolation of the findings. An interesting future study would be one that could quantitatively compare these three systems (in vitro, heterologous Drosophila and in vivo lepidoptera) to correlate their predictive abilities and design a standardized system for testing field-derived ABC transporter mutations.

## 4. Conclusions

In the present study, we designed transgenic *Drosophila* lines expressing FAW wildtype and mutant ABCC2 transporters. These fly lines can be employed as FAW ABCC2 models to investigate lepidopteran Bt toxin efficacy and resistance. Our results provide an efficient demonstration of the suitability of transgenic *Drosophila* for the validation of FAW ABCC2 resistance mutations in ECL4 against Bt toxins, and as a potential screening tool to find Bt toxins lacking cross-resistance.

## 5. Materials and Methods

### 5.1. Drosophila Strains

All fly strains used in this study are shown in Table S1. Strain VK13 was used for the integration of the transgenes at the attP insertion point on the 3rd chromosome via embryonic injections. The balancer line yw; TM3 Sb e/TM6B Tb Hu e was used for genetic crosses in order to generate homozygous lines. The driver lines, HR-GAL4—previously described [30]—MYO-GAL4 (RRID: BDSC_83278) and ELAV-GAL4 (RRID: BDSC_8760) were used in order to express the transgene in different tissues (for details refer to Table S1). HR-GAL4 carries GAL4 expression in the midgut, Malpighian tubules and fat body, ELAV-GAL4 drove expression in the brain and MYO-GAL4 drove expression specifically in the midgut. All flies were kept at a temperature of 25 °C, a humidity of 60–70% and a 12: 12 h photoperiod on a standard fly diet.

### 5.2. Generation of Constructs for Drosophila Transformation

The coding sequence of SfABCC2 (KY646296.1) was synthesized de novo (Life Technologies GmbH, Darmstadt, Germany) and modified to minimize DNA secondary structures and optimize codon usage for expression in *Drosophila*. The restriction sites of BstEII and AgeI were also introduced into the open reading frame (ORF) using synonymous sites, which allowed for downstream modifications. The 4067 bp length ORF was then cloned into the pUAST-attB vector. In order to introduce the GY deletion and the P799K substitution, alone and in combination, three fragments (488–489 bp length) were ordered as gBlocks from IDT (fragments’ sequences shown in Figure S1) with terminal BstEII and AgeI restriction sites. Each fragment was cloned into a pGEM T-easy vector (Promega, Madison, WI, USA). Subsequently, a BstEII/AgeI fragment encompassing the deletion and/or the substitution was cloned into pUAST-SfABCC2, which had been likewise digested with BstEII and AgeI so that the “wild type” sequence could be replaced by the mutant sequences. All plasmids were verified by sequencing (CEMIA). 

### 5.3. Generation of Transgenic Flies

We used the phiC31 integrase system to introduce the ABCC2 gene of *S. frugiperda* into the *Drosophila* genome. Embryos of the *D. melanogaster* strain VK13 were injected with the recombinant plasmids. An injection mix which consists of 100 ng/µL plasmid DNA in injection buffer (2 mM sodium phosphate, pH 6.8–7.8, 100 mM KCl) was used. Injected G0 flies were outcrossed with VK13 adult flies and the G1 progeny were screened for w+ phenotypes (orange eyes in the heterozygous state) indicating integration of the plasmid. Independent transformed lines were crossed with a balancer strain for the third chromosome (yw; TM3 Sb e/TM6B Tb Hu e), and G2 flies with orange eyes and the Sb phenotype were selected and crossed among themselves to generate homozygous flies (red eyes), used to establish the transgenic responder line population. In order to molecularly confirm the integration, PCR amplification was conducted for each fly line with diagnostic primer pairs (Table S2; Figure 1).

### 5.4. DNA Extraction and PCR Amplification

Genomic DNA was extracted from flies using DNAzol (MRC, Cincinnati, OH), following the manufacturer instructions. In order to confirm the integration of the transgenes, PCR amplification was performed with Minotech Taq DNA Polymerase (Minotech Biotechnology) and diagnostic primer pairs (Table S2; Figure 1). The conditions used were 94 °C for 2 min, followed by 30–35 cycles of denaturation at 94 °C for 45 s, annealing at 54–58 °C for 30 s and extension at 72 °C for 30–45 s, followed by a final extension step for 5 min.

### 5.5. Toxicity Bioassays

Xentari (*Bacillus thuringiensis* var. aizawai—Bta, strain ABTS-1857, 54% *w*/*w*, Valent Biosciences)—a commercially available Bt-based insecticide which is composed of Cry1Aa, Cry1Ab, Cry1C and Cry1D—was used in larval toxicity bioassays.

The bipartite GAL4/UAS system was used to drive the expression of the SfABCC2 transgene in specific tissues, which were then tested for toxicity. More precisely, males harboring a GAL4 construct were crossed to transgenic responder line virgin females containing a UAS-SfABCC2 variant created using the methods described above. The progeny of these crosses was used in feeding bioassays, as previously described [31]. In brief, HR-GAL4 males crossed with transgenic responder virgin females were put in cages to lay eggs on cherry juice agar for 24 h. First, instar larvae were collected and transferred in batches of 20 into new vials containing fly food supplemented with different Xentari concentrations. After a period of 10 days, the number of pupae were scored. Five to six insecticide concentrations that represented the full range of mortality (5–95%) in susceptible individuals were chosen and tested in triplicate, together with relevant negative (no insecticide) controls. Dose-dependent mortality curves were constructed from dose–response data, and LC_50_ values were calculated using PoloPlus 2.0 (LeOra Software LLC, Parma, MO, USA).

## Figures and Tables

**Table 1:** Log-dose probit-mortality for Xentari in larval bioassays with different strains of transgenic flies expressing SfABCC2 (wildtype and mutants).

Strain	LC_50_ (mg/L)	95% CI ^a^	Slope (±SE)	RR ^b^
VK13 × HR-GAL4 (no ABCC2)	>1000			
ABCC2 × HR-GAL4 (wildtype)	9.4	7.3–11.3	6.07 (1.24)	1
GYdel × HR-GAL4 (mutant)	27.1	24.1–30.2	5.35 (0.69)	2.88
P799K × HR-GAL4 (mutant)	44.1	32.7–51.6	6.01 (1.03)	4.69
Combined × HR-GAL4 (mutant)	143	127–171	5.22 (1.12)	15.2

^a^ Confidence interval, 95%; ^b^ Resistance ratio (LC_50_ of ABCC2 mutant strain divided by LC_50_ of ABCC2 wildtype strain).

## Data Availability

We will provide all data generated in this study upon request.

## Supplementary Materials

The following supporting information can be downloaded at: https://www.mdpi.com/article/10.3390/toxins15060386/s1, Figure S1: Sequences ordered as gBlocks to be used for the generation of the constructs for Drosophila transformation. The GY deletion is colored in green and the P799K substitution is colored in red; Table S1: Drosophila lines used in this study; Table S2: List of primers used in this study.

## References

[1] Overton K., Maino J.L., Day R., Umina P.A., Bett B., Carnovale D., Ekesi S., Meagher R., Reynolds O.L. (2021). Global Crop Impacts, Yield Losses and Action Thresholds for Fall Armyworm (*Spodoptera frugiperda*): A Review. Crop Prot..

[2] Sparks A.N. (1979). A Review of the Biology of the Fall Armyworm. Fla. Entomol..

[3] Tay W.T., Meagher R.L., Czepak C., Groot A.T. (2023). Spodoptera Frugiperda: Ecology, Evolution, and Management Options of an Invasive Species. Annu. Rev. Entomol..

[4] Kenis M., Benelli G., Biondi A., Calatayud P.-A., Day R., Desneux N., Harrison R.D., Kriticos D., Rwomushana I., van den Berg J. (2023). Invasiveness, Biology, Ecology, and Management of the Fall Armyworm, Spodoptera Frugiperda. Entomol. Gen..

[5] Ma J., Wang Y.-P., Wu M.-F., Gao B.-Y., Liu J., Lee G.-S., Otuka A., Hu G. (2019). High Risk of the Fall Armyworm Invading Japan and the Korean Peninsula via Overseas Migration. J. Appl. Entomol..

[6] Tepa-Yotto G.T., Tonnang H.E.Z., Goergen G., Subramanian S., Kimathi E., Abdel-Rahman E.M., Flø D., Thunes K.H., Fiaboe K.K.M., Niassy S. (2021). Global Habitat Suitability of Spodoptera Frugiperda (JE Smith) (Lepidoptera, Noctuidae): Key Parasitoids Considered for Its Biological Control. Insects.

[7] Zhou Y., Wu Q., Zhang H., Wu K. (2021). Spread of Invasive Migratory Pest Spodoptera Frugiperda and Management Practices throughout China. J. Integr. Agric..

[8] Sparks T.C., Crossthwaite A.J., Nauen R., Banba S., Cordova D., Earley F., Ebbinghaus-Kintscher U., Fujioka S., Hirao A., Karmon D. (2020). Insecticides, Biologics and Nematicides: Updates to IRAC’s Mode of Action Classification—A Tool for Resistance Management. Pestic. Biochem. Physiol..

[9] Adang M.J., Crickmore N., Jurat-Fuentes J.L., Dhadialla T.S., Gill S.S. (2014). Chapter Two-Diversity of Bacillus Thuringiensis Crystal Toxins and Mechanism of Action. Advances in Insect Physiology.

[10] Bravo A., Gill S.S., Soberón M. (2007). Mode of Action of Bacillus Thuringiensis Cry and Cyt Toxins and Their Potential for Insect Control. Toxicon.

[11] Heckel D.G. (2020). How Do Toxins from Bacillus Thuringiensis Kill Insects? An Evolutionary Perspective. Arch. Insect Biochem. Physiol..

[12] de Melo A.L.A., Soccol V.T., Soccol C.R. (2016). *Bacillus thuringiensis*: Mechanism of Action, Resistance, and New Applications: A Review. Crit. Rev. Biotechnol..

[13] Jurat-Fuentes J.L., Heckel D.G., Ferré J. (2021). Mechanisms of Resistance to Insecticidal Proteins from Bacillus Thuringiensis. Annu. Rev. Entomol..

[14] Endo H. (2022). Molecular and Kinetic Models for Pore Formation of Bacillus Thuringiensis Cry Toxin. Toxins.

[15] Heckel D.G. (2021). The Essential and Enigmatic Role of ABC Transporters in Bt Resistance of Noctuids and Other Insect Pests of Agriculture. Insects.

[16] Sato R., Adegawa S., Li X., Tanaka S., Endo H. (2019). Function and Role of ATP-Binding Cassette Transporters as Receptors for 3D-Cry Toxins. Toxins.

[17] Gahan L.J., Pauchet Y., Vogel H., Heckel D.G. (2010). An ABC Transporter Mutation Is Correlated with Insect Resistance to Bacillus Thuringiensis Cry1Ac Toxin. PLoS Genet..

[18] Jin M., Yang Y., Shan Y., Chakrabarty S., Cheng Y., Soberón M., Bravo A., Liu K., Wu K., Xiao Y. (2021). Two ABC Transporters Are Differentially Involved in the Toxicity of Two Bacillus Thuringiensis Cry1 Toxins to the Invasive Crop-Pest Spodoptera Frugiperda (J. E. Smith). Pest Manag. Sci..

[19] Banerjee R., Hasler J., Meagher R., Nagoshi R., Hietala L., Huang F., Narva K., Jurat-Fuentes J.L. (2017). Mechanism and DNA-Based Detection of Field-Evolved Resistance to Transgenic Bt Corn in Fall Armyworm (*Spodoptera frugiperda*). Sci. Rep..

[20] Flagel L., Lee Y.W., Wanjugi H., Swarup S., Brown A., Wang J., Kraft E., Greenplate J., Simmons J., Adams N. (2018). Mutational Disruption of the ABCC2 Gene in Fall Armyworm, Spodoptera Frugiperda, Confers Resistance to the Cry1Fa and Cry1A.105 Insecticidal Proteins. Sci. Rep..

[21] Boaventura D., Ulrich J., Lueke B., Bolzan A., Okuma D., Gutbrod O., Geibel S., Zeng Q., Dourado P.M., Martinelli S. (2020). Molecular Characterization of Cry1F Resistance in Fall Armyworm, Spodoptera Frugiperda from Brazil. Insect Biochem. Mol. Biol..

[22] Franz L., Raming K., Nauen R. (2022). Recombinant Expression of ABCC2 Variants Confirms the Importance of Mutations in Extracellular Loop 4 for Cry1F Resistance in Fall Armyworm. Toxins.

[23] Stevens T., Song S., Bruning J.B., Choo A., Baxter S.W. (2017). Expressing a Moth Abcc2 Gene in Transgenic Drosophila Causes Susceptibility to Bt Cry1Ac without Requiring a Cadherin-like Protein Receptor. Insect Biochem. Mol. Biol..

[24] Obata F., Tanaka S., Kashio S., Tsujimura H., Sato R., Miura M. (2015). Induction of Rapid and Selective Cell Necrosis in Drosophila Using Bacillus Thuringiensis Cry Toxin and Its Silkworm Receptor. BMC Biol..

[25] Denecke S., Swevers L., Douris V., Vontas J. (2018). How Do Oral Insecticidal Compounds Cross the Insect Midgut Epithelium?. Insect Biochem. Mol. Biol..

[26] Horikoshi R.J., Bernardi O., de Amaral F.S.A., Miraldo L.L., Durigan M.R., Bernardi D., Silva S.S., Omoto C. (2019). Lack of Relevant Cross-Resistance to Bt Insecticide XenTari in Strains of Spodoptera Frugiperda (J. E. Smith) Resistant to Bt Maize. J. Invertebr. Pathol..

[27] Douris V., Denecke S., Van Leeuwen T., Bass C., Nauen R., Vontas J. (2020). Using CRISPR/Cas9 Genome Modification to Understand the Genetic Basis of Insecticide Resistance: Drosophila and Beyond. Pestic. Biochem. Physiol..

[28] Samantsidis G.-R., Panteleri R., Denecke S., Kounadi S., Christou I., Nauen R., Douris V., Vontas J. (2020). “What I Cannot Create, I Do Not Understand”: Functionally Validated Synergism of Metabolic and Target Site Insecticide Resistance. Proc. Biol. Sci..

[29] Wang J., Wang H., Liu S., Liu L., Tay W.T., Walsh T.K., Yang Y., Wu Y. (2017). CRISPR/Cas9 Mediated Genome Editing of Helicoverpa Armigera with Mutations of an ABC Transporter Gene HaABCA2 Confers Resistance to Bacillus Thuringiensis Cry2A Toxins. Insect Biochem. Mol. Biol..

[30] Chung H., Bogwitz M.R., McCart C., Andrianopoulos A., ffrench-Constant R.H., Batterham P., Daborn P.J. (2007). Cis-Regulatory Elements in the Accord Retrotransposon Result in Tissue-Specific Expression of the Drosophila Melanogaster Insecticide Resistance Gene Cyp6g1. Genetics.

[31] Douris V., Steinbach D., Panteleri R., Livadaras I., Pickett J.A., Van Leeuwen T., Nauen R., Vontas J. (2016). Resistance Mutation Conserved between Insects and Mites Unravels the Benzoylurea Insecticide Mode of Action on Chitin Biosynthesis. Proc. Natl. Acad. Sci. USA.

