# Supplementary material for: Transgenic Drosophila to Functionally Validate Fall Armyworm ABCC2 Mutations Conferring Bt Resistance
Source: Toxins (Basel). 2023 Jun 7;15(6):386. doi: 10.3390/toxins15060386 (PMC10301211; doi:10.3390/toxins15060386)
Supplement: Supplementary file 1 [file toxins-15-00386-s001.zip › toxins-2426274-supplementary.pdf]

SUPPLEMENTARY MATERIAL

Table S1. *Drosophila* lines used in this study.

**Strain**	**Description**	**Source**
VK13	Expresses phiC31 integrase under the control of *vasa*. Contains an attP docking site in the 3rd chromosome.	IMBB flyroom stock
HR-GAL4	Bears HR-GAL4 transgene (marked with miniwhite) at P-element insertion site in 2nd chromosome. Drives the reporter gene expression in the larval midgut, Malpighian tubules and fat body.	Daborn et al., 2012
ELAV-GAL4	Neuronal cell-specific driver. Drives the reporter gene expression in embryonic glial cells and mitotically active cells.	IMBB flyroom stock
MYO-GAL4	Drives the expression in midgut cells.	
yw; TM3 *Sb e* / TM6B *Tb Hu e*	Balancers for 3rd chromosome (TM3 with Stubble marker. TM6B with Tubby and Humoral markers)	
UAS_ABCC2	Bears *SfABCC2* transgene under UAS at the attP insertion point at 3rd chromosome.	This study
UAS_ABCC2_GYdel	Bears *SfABCC2* transgene with the GY deletion at position 788-789 under UAS at the attP insertion point at 3rd chromosome.	This study
UAS_ABCC2_P799K	Bears *SfABCC2* transgene with the P799K substitution under UAS at the attP insertion point at 3rd chromosome.	This study
UAS_ABCC2_combo	Bears a *SfABCC2* transgene with the GY deletion and the P799K substitution under UAS at the attP insertion point at 3rd chromosome.	This study

Table S2. List of primers used in this study.

Primer name	5' sequence 3'	Use
ABCC2_F	GCAAGATGAGCGTGAAGTCC	Plasmid construction, sequencing
ABCC2_R	ATTCAGGACCAGCACGGAGG	
M13/pUC F	CCCAGTCACGACGTTGTAAAACG	Plasmid construction
M13/pUC R	AGCGGATAACAATTTCACACAGG	
ABCC2_Seq (F)	ATGCTAATGTGGGACGCCAG	Sequencing
ABCC2_WtDel	AACCAGGTGGACGGCTAC	Specific screen for integration
ABCC2_P799K	CCAGAGGGCGAGAGCAAG	
ABCC2_GYdel	GACCAACCAGGTGGACATTCAG	
3xP3_RFP_F	CCAACTGGGGTAACCTTTGA	Screen for integration
VK13_R	ATTCTTTCCGCTGATTGTGC	
SV40_F	CCCCCTGAACCTGAAACATA	

ABCC2_GYdel

GTACGGTTACCCACCAGATCCACTACCTGAAGGCCGCCGATTTCATCGTGGTGCTGA
ATGAGGGCAGCGTGGAAAACATGGGCAGCTACGATGAGCTGATGAAGACCGGCACCG
AGTTCTCCATGCTGTTGAGTGATCAGGCCAGCGAGGGCTCCGATACCGATAAGAAAG
AACGCCCAGCCATGATGCGCGGCATCAGCAAGATGAGCGTGAAGTCCGATGATGAAG
AGGGCGAAGAGAAGGTGCAGGTCCTGGAAGCCGAAGAACGTCAAAGCGGCAGCCTGA
AGTGGGATGTGTTGGGCCGCTACATGAAGTCCGTGAACTCGTGGTGTATGGTGGTCA
TGGCCTTTCTGGTGCTGGTCATTACACAGGGTGCCGCCACCACCACCGATTACTGGC
TGAGCTTCTGGACCAACCAGGTGGACATTCAGACACTGCCAGAGGGCGAGAGCCCCA
ATCCAGAGTTGAATACCCAAGTGGGCCTGCTGACCACCGGTCGTAC

ABCC2_P799K

GTACGGTTACCCACCAGATCCACTACCTGAAGGCCGCCGATTTCATCGTGGTGCTGA
ATGAGGGCAGCGTGGAAAACATGGGCAGCTACGATGAGCTGATGAAGACCGGCACCG
AGTTCTCCATGCTGTTGAGTGATCAGGCCAGCGAGGGCTCCGATACCGATAAGAAAG
AACGCCCAGCCATGATGCGCGGCATCAGCAAGATGAGCGTGAAGTCCGATGATGAAG
AGGGCGAAGAGAAGGTGCAGGTCCTGGAAGCCGAAGAACGTCAAAGCGGCAGCCTGA
AGTGGGATGTGTTGGGCCGCTACATGAAGTCCGTGAACTCGTGGTGTATGGTGGTCA
TGGCCTTTCTGGTGCTGGTCATTACACAGGGTGCCGCCACCACCACCGATTACTGGC
TGAGCTTCTGGACCAACCAGGTGGACGGCTACATTCAGACACTGCCAGAGGGCGAGA
GCAAGAATCCAGAGTTGAATACCCAAGTGGGCCTGCTGACCACCGGTCGTAC

ABCC2_GYdel_P799K

GTACGGTTACCCACCAGATCCACTACCTGAAGGCCGCCGATTTCATCGTGGTGCTGA
ATGAGGGCAGCGTGGAAAACATGGGCAGCTACGATGAGCTGATGAAGACCGGCACCG
AGTTCTCCATGCTGTTGAGTGATCAGGCCAGCGAGGGCTCCGATACCGATAAGAAAG
AACGCCCAGCCATGATGCGCGGCATCAGCAAGATGAGCGTGAAGTCCGATGATGAAG
AGGGCGAAGAGAAGGTGCAGGTCCTGGAAGCCGAAGAACGTCAAAGCGGCAGCCTGA
AGTGGGATGTGTTGGGCCGCTACATGAAGTCCGTGAACTCGTGGTGTATGGTGGTCA
TGGCCTTTCTGGTGCTGGTCATTACACAGGGTGCCGCCACCACCACCGATTACTGGC
TGAGCTTCTGGACCAACCAGGTGGACATTCAGACACTGCCAGAGGGCGAGAGCAAGA
ATCCAGAGTTGAATACCCAAGTGGGCCTGCTGACCACCGGTCGTAC

Figure S1. Sequences ordered as gBlocks to be used for the generation of the constructs for *Drosophila* transformation. The GY deletion is colored with green and the P799K substitution is colored with red.
